# The impact of thrombocytopenia on prognosis of HBV-related small hepatocellular carcinoma: a propensity score matching analysis

**DOI:** 10.1186/s12957-021-02160-2

**Published:** 2021-02-11

**Authors:** Wei Peng, Chuan Li, Xiaoyun Zhang, Tianfu Wen, Zheyu Chen

**Affiliations:** Department of Liver Surgery & Liver Transplantation Center, West China Hospital, Sichuan University, Chengdu, 610041 China

**Keywords:** Hepatocellular carcinoma, Liver resection, Platelet, Thrombocytopenia

## Abstract

**Background:**

Thrombocytopenia was reported both detrimental and advantageous to hepatocellular carcinoma (HCC). However, there is little evidence showing clearly the clinical value of preoperative thrombocytopenia on the surgical outcome of patients with small HCC. This retrospective study aimed at elucidating the correlation between preoperative thrombocytopenia and surgical outcome of small HCC patients within Milan criteria treated with liver resection.

**Methods:**

Data of hepatitis B virus (HBV)-related small HCC patients were retrospectively analyzed, and we performed the propensity score matching (PSM) analysis to overcome the imbalance of clinicopathological features. Patients enrolled were subsequently categorized into two groups according to preoperative platelet counts: thrombocytopenia group and non-thrombocytopenia group. Survival outcomes of the patients in both groups were described with the Kaplan-Meier method, and the difference was compared with a log-rank test. Cox regression analysis was applied to identify the risk factors of surgical outcome.

**Results:**

After PSM, the estimated 1-, 3-, and 5-year overall survival (OS) rates for small HCC patients in the thrombocytopenia group were 94.5%, 77.0%, and 57.6%, and 95.0%, 79.6%, and 68.0%, respectively, for small HCC patients in the non-thrombocytopenia group (*P* = 0.042). And the 1-, 3-, and 5-year estimated recurrence-free survival (RFS) rates for small HCC patients in the thrombocytopenia group were 70.4%, 51.0%, and 42.1%, and 83.8%, 63.7%, and 46.7%, respectively, for small HCC patients in the non-thrombocytopenia group (*P* = 0.035). Multivariate analysis indicated preoperative thrombocytopenia was a significant prognosticator of poor RFS (hazard ratio (HR) = 1.388, 95% confidence interval (CI) 1.028~1.874, *P* = 0.033).

**Conclusion:**

Preoperative thrombocytopenia had an undesirable impact on the recurrence of small HCC patients treated with liver resection.

**Supplementary Information:**

The online version contains supplementary material available at 10.1186/s12957-021-02160-2.

## Background

Hepatocellular carcinoma (HCC) is reported to be a common malignancy with high rates of incidence and mortality [1]. Nearly 50% of the newly diagnosed cases and HCC-related deaths existed in China due to the large population and heavy burden of hepatitis B virus (HBV) infection [2]. Patients with small HCC meeting the Milan criteria which is a special subgroup of HCC are supposed to have a better prognosis than those beyond the Milan criteria [3]. Liver resection is reported to be the optimal choice for small HCC [4]. However, survival remains unsatisfactory after liver resection due to the high recurrence rate [5]. Continuous effort to predict recurrence following liver resection is needed.

Plenty of evidence showed platelets had multiple functions other than hemostasis and that they were also involved in inflammation, immunity, regeneration, and carcinogenesis of the liver [6]. The platelets’ adhesion to tumor cells may form a “shield” which protects tumor cells from immune clearance [7]. Clinical data revealed that thrombocytosis was related to a worse prognosis in several malignancies [8, 9]. Prophylactic antiplatelet therapy was reported to reduce hepatocarcinogenesis in cirrhotic patients and decrease recurrence in HCC patients [10–12].

Thrombocytopenia, a symbol of liver cirrhosis and portal hypertension, has been reported to be associated with posthepatectomy liver failure (PHLF) and perioperative deaths [13]. Nevertheless, the clinical impact of thrombocytopenia on the long-term survival of HCC patients remains controversial. On one side, several studies have indicated that preoperative thrombocytopenia was a valuable predictor of recurrence and death for HCC patients [14–17]. On the other side, some recent studies found that thrombocytopenia was associated with better survival in patients with advanced HCC [18–21]. Furthermore, to our knowledge, there is no evidence showing the clinical value of thrombocytopenia on the long-term outcome of HBV-related small HCC patients within the Milan criteria.

The present retrospective study was performed to elucidate the clinical impact of thrombocytopenia on the surgical outcome of HBV-related small HCC patients.

## Methods

This study was carried out with the approval of the Ethics Committee of our institute according to the Helsinki Declaration of 1975. Clinical data of HBV-related small HCC patients treated with liver resection between February 2007 and January 2016 in our center was collected from our prospective database. Histopathological characteristics including tumor differentiation, microvascular invasion (MVI), and liver cirrhosis were assessed by a hepatologist. In the current study, thrombocytopenia was defined as a platelet count less than 100 × 10^9^/L according to a previous report [22].

The inclusion criteria of the present study were as follows: (1) pathologically confirmed HCC, (2) tumor within the Milan criteria, (3) treated with liver resection as the initial treatment, (4) normal liver function: Child-Pugh class A, and (5) positive HBV surface antigen. The exclusion criteria are as follows: (1) extrahepatic malignancies and (2) poor data integrity.

### Follow-up

The follow-up strategy was in accordance with the consensus we previously reported. Each patient was routinely followed up at the first month after surgery, trimonthly in the upcoming 3 years, and every 6 months subsequently [23]. Complete blood cell, liver function, concentration of alpha-fetoprotein (AFP), HBV-DNA level, and imaging examination should be included in each follow-up visit. Antiviral therapy would be initiated to patients according to the guideline suggestion. Recurrent HCC was diagnosed when typical features of HCC were revealed by two types of imaging examination or specific image finding along with an elevated AFP concentration. The final follow-up visit occurred on June 30, 2016.

### Statistical analysis

Categorical data were displayed as numbers (percentage) and compared using the *χ*^2^ test, while the continuous variables were displayed as mean value ± standard deviation and compared by the independent sample *t* test. Survival outcomes in different groups were analyzed by the Kaplan-Meier method and were compared by a log-rank test. Potential variables with a *P* value of less than 0.05 in the univariate analysis were subsequently tested for proportional hazard assumption. Multivariate Cox regression analysis was used to investigate significant predictors for both overall survival (OS) and recurrence-free survival (RFS).

In this study, a PSM analysis was implemented to balance the bias of covariates. A multivariable logistic regression model was built to predict the probability of each patient being assigned to each group based on a set of known co-variables possibly affecting postoperative outcomes: age, gender, AFP level, liver function indicators, tumor size and number, liver cirrhosis, MVI, tumor differentiation, and transfusion. The predicted values were then used to obtain 1:1 nearest-neighbor matching with a caliper value of 0.2. The nearest-neighbor matching was selected by matching the subject in the thrombocytopenia group to whose propensity score is closest to that of the subject in the non-thrombocytopenia group. Comparisons after propensity matching were calculated in terms of standardized mean difference. A difference less than 20% of absolute value was considered to be acceptable. Patients for whom the propensity score could not be matched exactly were excluded. Univariate and multivariate analyses were based on the data after PSM.

Statistics were accomplished by the SPSS software for Windows (version 25.0, IBM Corp, Armonk, NY, USA) and R software version 4.0.0 (R Foundation for Statistical Computing, Vienna, Austria; http://www.r-project.org). The R package of Survival was used for the proportional hazard assumption, and Jon Peck extension for PSM (version 1.5.0, IBM Corp, Armonk, NY, USA) was used for PSM analysis.

## Results

### Patient features

The present study enrolled as many as 582 HBV-related small HCC patients who underwent liver resection. PLT of the patients in the whole study ranged from 19 × 10^9^/L to 469 × 10^9^/L; among them, 247 (42.4%) had a preoperative platelet count of <100 × 10^9^/L and were categorized as the thrombocytopenia group, while 335 (57.6%) had a preoperative platelet count of ≥100 × 10^9^/L and were categorized as the non-thrombocytopenia group. Table 1 shows the detailed clinicopathologic features of patients in these two groups. The comparison of several indicators of the liver function between the two groups revealed that patients in the non-thrombocytopenia group exhibited a lower incidence of underlying liver cirrhosis, lower total bilirubin, higher serum albumin, and shorter prothrombin time (*P* < 0.05). Nevertheless, patients from the thrombocytopenia group had smaller but multiple tumors (*P* < 0.05). After the PSM, 201 matched pairs were created for further analysis. The baseline features were comparable between those two matched cohorts (Table 1).

**Table 1:** Baseline features of two groups based on preoperative platelet counts

Variables	Whole study population			Propensity score-matched pairs		
	PLT < 100 (×10^9^/L), *n* = 247	PLT ≥ 100 (×10^9^/L), *n* = 335	*P*	PLT < 100 (×10^9^/L), *n* = 201	PLT ≥ 100 (×10^9^/L), *n* = 201	*P*
Age (years)	51.18 ± 11.04	49.36 ± 11.37	0.054	50.25 ± 11.38	50.06 ± 12.07	0.872
Male/female	207/40	288/47	0.482	173/28	164/37	0.278
AFP > 400 ng/mL	76 (30.8%)	100 (29.9%)	0.855	62 (30.8%)	63 (31.3%)	1.000
TBIL (μmol/L)	16.65 ± 6.70	14.33 ± 6.00	< 0.001	15.61 ± 5.51	15.57 ± 6.71	0.941
ALB (g/L)	41.53 ± 4.40	42.52 ± 5.03	0.014	42.15 ± 3.99	42.11 ± 4.41	0.921
ALT (IU/L)	47.82 ± 35.86	47.20 ± 46.75	0.862	48.44 ± 37.47	45.96 ± 42.20	0.533
AST (IU/L)	42.45 ± 25.12	39.48 ± 39.64	0.302	41.19 ± 25.29	39.38 ± 35.53	0.556
PT (s)	12.43 ± 1.20	12.02 ± 1.09	< 0.001	12.25 ± 1.14	12.17 ± 1.13	0.517
CREA (μmol/L)	77.23 ± 24.14	75.94 ± 14.13	0.422	77.11 ± 15.15	75.60 ± 15.28	0.318
Tumor size < 3 cm	140 (56.9%)	160 (47.8%)	0.036	106 (52.7%)	112 (55.7%)	0.617
Solitary tumor	212 (85.8%)	308 (91.9%)	0.021	180 (89.6%)	184 (91.5%)	0.610
Transfusion (+/−)	26/221	10/325	< 0.001	10/191	10/191	1.000
Cirrhosis (+/−)	231/16	270/65	< 0.001	186/15	187/14	1.000
MVI (+/−)	49/198	47/288	0.071	40/161	37/164	0.800
Poor differentiation	99 (40.1%)	113 (33.7%)	0.117	84 (41.8%)	79 (39.3%)	0.685

*PLT* platelet counts, *AFP* alpha-fetoprotein, *TBIL* total bilirubin, *ALB* albumin, *ALT* alanine aminotransferase, *AST* aspartate aminotransferase, *PT* prothrombin time, *CREA* creatinine, *MVI* microvascular invasion

### Impact of thrombocytopenia on survival

**Fig. 1:**
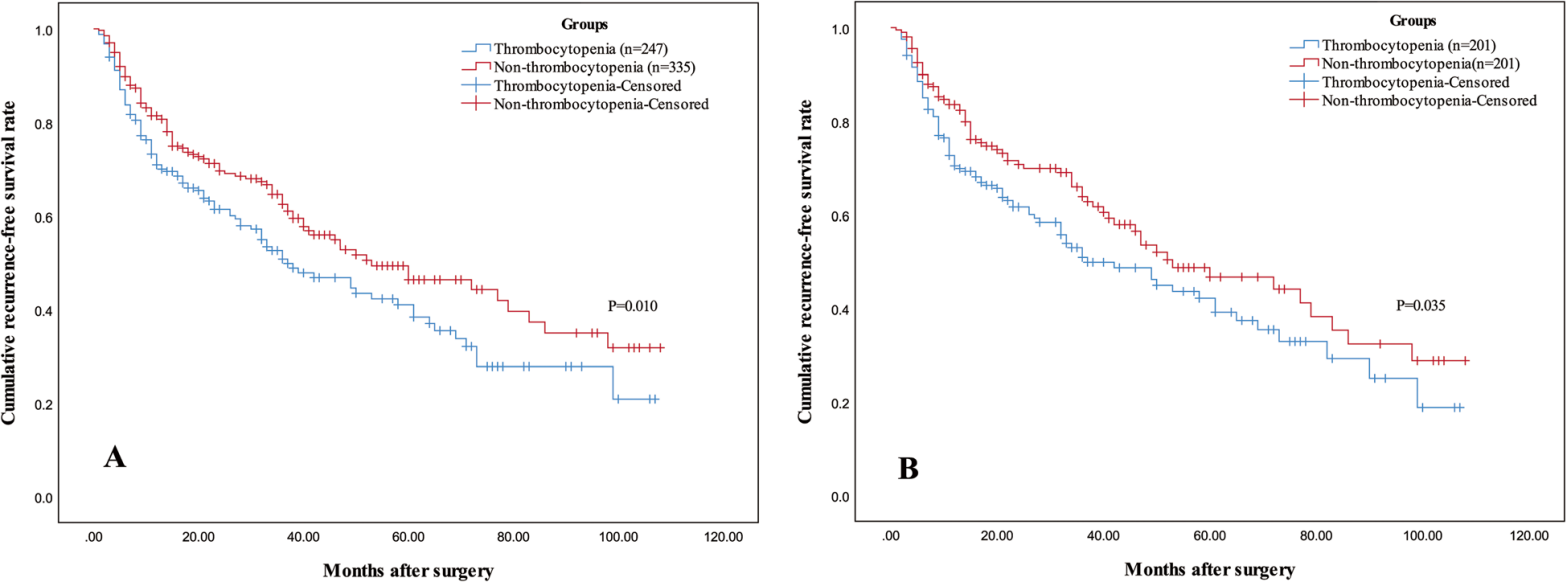
Kaplan-Meier curves of RFS for small HCC patients in the thrombocytopenia and non-thrombocytopenia groups before (**a**) and after (**b**) PSM. Patients in the thrombocytopenia group had decreased recurrence-free survival before (**a**) and after (**b**) PSM. RFS, recurrence-free survival; HCC, hepatocellular carcinoma; PSM, propensity score matching

After 35 months of median follow-up time, 241 (41.4%) patients were found recurrent and 148 (25.4%) patients died. Before PSM, 1-, 3-, and 5-year estimated OS rates of patients in the thrombocytopenia group were 93.3%, 74.9%, and 55.5%, separately, and 95.1%, 77.6%, and 68.2%, separately, for patients in the non-thrombocytopenia group, respectively (*P* = 0.029). One-, 3-, and 5-year estimated RFS rates of patients in the thrombocytopenia group were 71.1%, 50.9%, and 41.4%, separately, and 81.1%, 62.7%, and 50.0%, separately, for patients in the non-thrombocytopenia group, respectively (*P* = 0.010, Fig. 1a).

After PSM, 1-, 3-, and 5-year estimated OS rates of patients in the thrombocytopenia group were 94.5%, 77.0%, and 57.6%, separately, and 95.0%, 79.6%, and 68.0%, separately, for patients in the non-thrombocytopenia group, respectively (*P* = 0.042). One-, 3-, and 5-year estimated RFS rates of patients in the thrombocytopenia group were 70.4%, 51.0%, and 42.1%, separately, and 83.8%, 63.7%, and 46.7%, separately, for patients in the non-thrombocytopenia group, respectively (*P* = 0.035, Fig. 1b).

### Independent risk factors of prognosis

After PSM, univariate analysis suggested that creatinine (*P* = 0.012), presence of MVI (*P* < 0.001), poor differentiation (*P* < 0.001), and thrombocytopenia (*P* = 0.042) were related to reduced OS. These four covariates all meet proportional hazard assumption (Supplementary Table 1). Cox regression analysis suggested that elevated creatinine (HR = 1.014, 95% CI 1.002~1.027, *P* = 0.025) and presence of MVI (HR = 2.628, 95% CI 1.759~3.925, *P* < 0.001) were prognostic factors for reduced OS (Table 2). Univariate analysis found poor differentiation (*P* < 0.001), multiple tumors (*P* = 0.004), presence of MVI (*P* < 0.001), and thrombocytopenia (*P* < 0.001) were significantly related to recurrence. These four covariates all meet proportional hazard assumption (Supplementary Table 1). Then, Cox regression suggested poor differentiation (HR = 1.399, 95% CI 1.024~1.911, *P* = 0.035), multiple tumors (HR = 2.561, 95% CI 1.658~3.955, *P* < 0.001), presence of MVI (HR = 1.970, 95% CI 1.407~2.757, *P* < 0.001), and thrombocytopenia (HR = 1.388, 95% CI 1.028~1.874, *P* = 0.033) were found to be prognostic factors for reduced RFS (Table 3).

**Table 2:** Prognostic factors of overall survival in propensity score-matched pairs

Factors	Univariate analysis		Multivariate analysis	
	HR (95% CI)	*P*	HR (95% CI)	*P*
Age	0.996 (0.977~1.015)	0.657		
Gender (male)	1.709 (0.874~3.340)	0.117		
TBIL	0.996 (0.961~1.033)	0.844		
ALT	1.004 (0.999~1.009)	0.160		
AST	1.002 (0.996~1.009)	0.484		
ALB	0.969 (0.920~1.021)	0.241		
PT	0.969 (0.795~1.181)	0.757		
CREA	1.019 (1.004~1.034)	0.012	1.014 (1.002~1.027)	0.025
AFP (> 400 ng/mL)	1.421 (0.890~2.269)	0.141		
Tumor size (3~5 cm)	1.326 (0.850~2.069)	0.214		
Tumor number (2~3)	2.214 (1.114~4.399)	0.023		
Transfusion (+)	1.539 (0.597~3.968)	0.372		
Cirrhosis (+)	1.136 (0.471~2.741)	0.777		
MVI (+)	3.432 (2.040~5.773)	< 0.001	2.628 (1.759~3.925)	< 0.001
Poor differentiation (+)	3.579 (2.251~5.691)	< 0.001		
PLT (< 100 × 10^9^/L)	1.592 (1.016~2.493)	0.042		

*HR* hazard ratio, *CI* confidence interval, *TBIL* total bilirubin, *ALT* alanine aminotransferase, *AST* aspartate aminotransferase, *ALB* albumin, *PT* prothrombin time, *CREA* creatinine, *AFP* alpha-fetoprotein, *MVI* microvascular invasion, *PLT* platelet counts

**Table 3:** Prognostic factors of recurrence-free survival in propensity score-matched pairs

Factors	Univariate analysis		Multivariate analysis	
	HR (95% CI)	*P*	HR (95% CI)	*P*
Age	0.993 (0.976~1.010)	0.421		
Gender (male)	1.587 (0.909~2.769)	0.104		
TBIL	0.995 (0.963~1.028)	0.758		
ALT	1.003 (0.998~1.008)	0.199		
AST	1.006 (0.999~1.013)	0.111		
ALB	0.963 (0.918~1.009)	0.116		
PT	0.894 (0.748~1.068)	0.215		
CREA	1.007 (0.994~1.020)	0.300		
AFP (> 400 ng/mL)	1.163 (0.760~1.780)	0.485		
Tumor size (3~5 cm)	1.077 (0.725~1.601)	0.714		
Tumor number (2~3)	2.807 (1.391~5.664)	0.004	2.561 (1.658~3.955)	< 0.001
Transfusion (+)	0.877 (0.350~2.194)	0.779		
Cirrhosis (+)	1.744 (0.774~3.931)	0.180		
MVI (+)	2.837 (1.694~4.750)	< 0.001	1.970 (1.407~2.757)	< 0.001
Poor differentiation (+)	3.778 (2.483~5.750)	< 0.001	1.399 (1.024~1.911)	0.035
PLT (< 100 × 10^9^/L)	1.534 (1.031~2.282)	0.035	1.388 (1.028~1.874)	0.033

*HR* hazard ratio, *CI* confidence interval, *TBIL* total bilirubin, *ALT* alanine aminotransferase, *AST* aspartate aminotransferase, *ALB* albumin, *PT* prothrombin time, *CREA* creatinine, *AFP* alpha-fetoprotein, *MVI* microvascular invasion, *PLT* platelet counts

## Discussion

Much attention has been given to the prognostic value of platelets, which has the advantage of being readily available from routine test of blood cells. Thrombocytopenia per se was reported to cause different outcomes in HCC patients. The results of this retrospective study demonstrated preoperative thrombocytopenia a reliable prognosticator of decreased RFS for small HCC.

Bihari et al. reported the association of platelets with HCC after analyzing consecutive 1008 cirrhosis and 420 HCC cases [18]. In their study, platelet counts increased after carcinogenesis from cirrhosis and decreased after treatment, and this phenomenon indicated that platelets may be stimulated by the HCC-related factors. Both systemic inflammatory response and HCC followed by chronic liver disease are often associated with elevated interleukin-6 (IL-6) levels which stimulates thrombopoiesis through thrombopoietin [24]. In other words, HCC would thus induce the increase of platelet counts. Platelets play a significant part in inflammation, fibrogenesis, and oncogenesis of the liver [6]. In a study on mice infected by HBV, researchers pointed out that platelets took a part in the etiopathogenesis of HCC [25]. Platelets facilitate invasion and metastasis of cancers by the formation of platelet coating shielding cancer cells and evading immune detection [26]. Increase of angiogenesis factors initiated by platelet activation would also promote tumor growth [27].

Thrombocytopenia is reported to be the most common hematologic complication in cirrhotic patients [28]. Decreased thrombopoietin production in the liver and increased platelet sequestration in the spleen followed by liver cirrhosis and hypersplenism were the major mechanisms of thrombocytopenia [22, 29]. A previous research also reported that degrees of impaired hepatic function may vary even in the same Child-Pugh class [30]. In line with that, we observed that patients in the thrombocytopenia group exhibited worse liver function reserve and higher incidence of cirrhosis (Table 1). Previous studies have confirmed that both liver cirrhosis and liver function reserve were strong predictors for postoperative recurrence of HCC [31, 32]. It was confirmed in the present study that patients in the thrombocytopenia group had an increased risk of postoperative recurrence. Interestingly, we also found patients in the thrombocytopenia group trended to have smaller but more tumors (*P* < 0.05), and tumor number (2~3) but not tumor size was found to be an independent risk factor of recurrence in Cox regression. Kubo and his colleagues also found a strong correlation between thrombocytopenia and multicentricity of HCC [33]. Thrombocytopenia caused by extensive fibrosis/cirrhosis which was related to worse liver function reserve and multicentricity of HCC might promote tumor recurrence after liver resection.

Nouso et al. revealed thrombocytopenia a significant risk factor of intrahepatic distant recurrence for HCC patients who underwent local ablation therapies [34]. He proposed that multicentric occurrence of HCC served as a feasible explanation for the close correlation between intrahepatic recurrence and underlying hepatic factors including thrombocytopenia. Similarly, thrombocytopenia was reported to be related to poor OS after radiofrequency ablation for HCC [16]. Moreover, Amano et al. found that thrombocytopenia predicted postoperative death and recurrence for HCC patients exceeding the Milan criteria [14]. Besides, results from two western centers revealed that a pretreatment platelet count less than 150 × 10^9^/L was a significant prognosticator of poor surgical outcome for patients with HCC ≤ 2 cm [15]. In the present study, we used a PSM analysis to balance the bias between the two groups and found preoperative thrombocytopenia an independent risk factor of RFS for HBV-related small HCC within the Milan criteria treated with liver resection. Patients included in the abovementioned studies shared a common character of early-stage HCC. Thrombocytopenia, the common complication of cirrhosis plays a negative impact on the prognosis of early-stage HCC.

However, some other studies found that HCC patients accompanied with thrombocytopenia had favorable outcomes. Morimoto and his colleagues found that HCC patients accompanied with thrombocytopenia were at lower risk of extrahepatic metastasis [19]. In their study, high platelet counts were significantly associated with the presence of portal vein tumor thrombus (PVTT) which was a strong predictor of extrahepatic metastasis. Xue et al. reported a similar result that high platelet counts increased the risk of extrahepatic metastasis of huge HCC treated with transcatheter arterial chemoembolization (TACE) [35]. Recently, Cheng et al. reported that preoperative thrombocytopenia was closely related to a favorable outcome of HCC patients with PVTT treated with liver resection [20]. Furthermore, several studies suggested that antiplatelet treatment helped to decrease the risk of hepatocarcinogenesis and recurrence of HCC [10, 11, 36]. Antiplatelet therapy served as an anti-HCC agent via decreasing epithelial angiogenesis and modulating systemic inflammation [37, 38].

We attempted to compromise the paradox of thrombocytopenia being both detrimental and advantageous to HCC. The pathophysiology of thrombocytopenia in HCC patients is complex since portal hypertension and hypersplenism followed by liver cirrhosis would lead to reduced platelet counts while systematic inflammation driven by HCC would increase platelet counts [39]. We hypothesized that biological behavior of tumor or liver function reserve would play a decisive role in the prognosis of HCC in different stages of HCC. Liver function reserve plays the decisive role in the prognosis of early-stage HCC, while the biological behavior of early-stage HCC is less malignant. HCC patients with thrombocytopenia which indicates worse liver function reserve would thus have a worse prognosis. On the other side, biological behavior plays the decisive role in prognosis of advanced-stage HCC, HCC patients with thrombocytopenia which reflected less thrombopoietin and IL-6 induced by less malignant tumor would have a better prognosis. This hypothesis helps to explain why studies based on different patient populations reached different conclusions. Studies [15–17, 34] that enrolled early-stage HCC patients who received liver resection or ablation suggested that thrombocytopenia was associated with poor prognosis, while studies [19, 20, 35] that enrolled advanced-stage HCC patients who received TACE or liver section suggested thrombocytopenia was associated with favorable prognosis.

Several limitations exist in this study. First, HCC patients enrolled in the present study are HBV-related, while most HCC cases are related to hepatitis C virus and alcohol in Western countries. So, the results of the present study should be further validated by a different etiological population. Second, there might be potential selection bias even though we used PSM analysis to balance the bias due to the mono-center, retrospective nature of this study.

## Conclusions

In conclusion, the present study found that preoperative thrombocytopenia had an undesirable impact on postoperative recurrence of HBV-related small HCC patients.

## Supplementary Information

**Additional file 1: Table 1.** Results of proportional hazard assumption before Cox regression for OS and RFS.

## Data Availability

The datasets generated and/or analyzed during the current study are not publicly available due to patient privacy but are available from the corresponding author on reasonable request.

## Abbreviations

HCC: Hepatocellular carcinoma
PSM: Propensity score matching
OS: Overall survival
RFS: Recurrence-free survival
HBV: Hepatitis B virus
PHLF: Posthepatectomy liver failure
MVI: Microvascular invasion
AFP: Alpha-fetoprotein
IL-6: Interleukin-6
PVTT: Portal vein tumor thrombus
