# Supplementary material for: The impact of thrombocytopenia on prognosis of HBV-related small hepatocellular carcinoma: a propensity score matching analysis
Source: World J Surg Oncol. 2021 Feb 11;19:46. doi: 10.1186/s12957-021-02160-2 (PMC7879633; doi:10.1186/s12957-021-02160-2)
Supplement: Supplementary file 1 — Additional file 1: Table 1. Results of proportional hazard assumption before Cox regression for OS and RFS. [file 12957_2021_2160_MOESM1_ESM.docx]

Supplementary Table 1. Results of proportional hazard assumption before Cox regression for OS and RFS.

| Covariates | OS | | RFS | |
| --- | --- | --- | --- | --- |
|  | χ² | P | χ² | P |
| CREA | 0.242 | 0.623 | NA | NA |
| Tumor number (2~3) | 0.532 | 0.466 | 0.210 | 0.651 |
| Poor differentiation (+) | 1.425 | 0.372 | 0.429 | 0.512 |
| MVI (+) | 0.255 | 0.614 | 2.202 | 0.142 |
| PLT (<100*10^9/L) | 0.151 | 0.698 | 2.278 | 0.134 |

CREA=creatinine; MVI=microvascular invasion; PLT=platelet counts; OS=overall survival; RFS=recurrence free survival; NA=not applicable.
